# The ArgR-Regulated ADI Pathway Facilitates the Survival of *Vibrio fluvialis* under Acidic Conditions

**DOI:** 10.3390/ijms25115679

**Published:** 2024-05-23

**Authors:** Qian Cheng, Yu Han, Yue Xiao, Zhe Li, Aiping Qin, Saisen Ji, Biao Kan, Weili Liang

**Affiliations:** National Key Laboratory of Intelligent Tracking and Forecasting for Infectious Diseases, National Institute for Communicable Disease Control and Prevention, Chinese Center for Disease Control and Prevention, Beijing 102206, China

**Keywords:** arginine deiminase (ADI) pathway, ArgR, *Vibrio fluvialis*, acidic conditions, regulation

## Abstract

*Vibrio fluvialis* is an emerging foodborne pathogenic bacterium that can cause severe cholera-like diarrhea and various extraintestinal infections, posing challenges to public health and food safety worldwide. The arginine deiminase (ADI) pathway plays an important role in bacterial environmental adaptation and pathogenicity. However, the biological functions and regulatory mechanisms of the pathway in *V. fluvialis* remain unclear. In this study, we demonstrate that L-arginine upregulates the expression of the ADI gene cluster and promotes the growth of *V. fluvialis*. The ADI gene cluster, which we proved to be comprised of two operons, *arcD* and *arcACB*, significantly enhances the survival of *V. fluvialis* in acidic environments both in vitro (in culture medium and in macrophage) and in vivo (in mice). The mRNA level and reporter gene fusion analyses revealed that ArgR, a transcriptional factor, is necessary for the activation of both *arcD* and *arcACB* transcriptions. Bioinformatic analysis predicted the existence of multiple potential ArgR binding sites at the *arcD* and *arcACB* promoter regions that were further confirmed by electrophoretic mobility shift assay, DNase I footprinting, or point mutation analyses. Together, our study provides insights into the important role of the ArgR-ADI pathway in the survival of *V. fluvialis* under acidic conditions and the detailed molecular mechanism. These findings will deepen our understanding of how environmental changes and gene expression interact to facilitate bacterial adaptations and virulence.

## 1. Introduction

During evolution, bacterial pathogens have developed different strategies to adapt to diverse environmental stresses and resist host immune defenses [1,2]. These defense strategies enable bacteria to survive infections and exploit nutrient-rich environments in diverse hosts to enhance their survival [1,3,4,5]. Arginine, a semi-essential amino acid, produces the antimicrobial metabolite nitric oxide (NO), which plays a crucial role in both innate and adaptive immune responses [6,7]. In bacterial pathogens, arginine can be metabolized by various pathways [8,9,10,11,12], among which the arginine deaminase (ADI) pathway, which is widely observed in microorganisms, helps bacteria adapt to hostile ecological niches and evade host defenses [13,14,15,16,17,18,19].

The ADI pathway is essential for bacterial survival under acidic conditions [12,20,21,22,23]. In this pathway, arginine is metabolized to generate ATP, carbon dioxide, and ammonia. As a metabolite of arginine, ammonia produces NH_4_^+^, which increases the pH of the cytoplasm and thus protects the bacteria from being killed by hostile acidic conditions [20,23]. In addition, another metabolite, ATP, provides energy to bacteria and facilitates the translocation of protons within the cytoplasm, preserving the homeostasis of cytosolic pH [20,24]. Moreover, the ADI pathway plays an important role in bacterial virulence and resistance to environmental stress [20,22,25,26]. In *Salmonella typhimurium*, the ADI pathway has been identified as a virulence factor because deletion of the ADI gene significantly reduces bacterial replication in murine macrophages and attenuates bacterial survival in mouse models [25]. ADI is also required for the survival of *Listeria monocytogenes* within macrophages and in the spleen of mouse models [20].

The ADI pathway is a crucial multienzyme pathway primarily composed of *arc* operons, including *arcA* (arginine deaminase), *arcB* (ornithine carbamoyltransferase), and *arcC* (carbamate kinase). Many ADI pathways also involve additional genes encoding arginine-ornithine antiporter (*arcD*) and a putative aminopeptidase [20,22]. The expression of the ADI pathway is tightly regulated by various environmental stresses, including carbon catabolite repression [27,28], low pH [17,29], anaerobiosis [14,30], temperature [31], and extracellular L-arginine levels [14,19,22]. In addition, the transcription factor ArgR, which is independent of the *arc* operons, significantly contributes to the regulation of the ADI pathway [14,18,20,22,32]. ArgR belongs to the ArgR/AhrC family of transcriptional regulators and normally binds to specific DNA sequences in the promoter region, known as the ARG box, through a conserved DNA binding motif at the N-terminus to exert regulatory effects [33,34,35]. ArgR in *Escherichia coli* forms a hexamer through two trimers, binding to an incomplete palindromic 18-nucleotide ARG box with two tandem repeats. In contrast, in *Bacillus licheniformis* [36] and *Streptococcus suis* [22], the binding site may only have one repeat. The pattern of ArgR binding varies in different bacteria.

*Vibrio fluvialis* is a saline, Gram-negative, facultative anaerobic bacterium commonly found in rivers and coastal waters. It is classified as a novel foodborne enteric pathogen that causes cholera-like acute diarrhea and a variety of extraintestinal infections [37,38,39,40]. Outbreaks of acute gastroenteritis resulting from *V. fluvialis* infection have been reported worldwide. In addition, *V. fluvialis* infects a range of fishes and aquatic animals, resulting in considerable economic losses in the aquaculture industry. Therefore, *V. fluvialis* is considered an emerging threat to public health, food safety, and economic development.

ADI positivity is one of the crucial biochemical characteristics that distinguishes *V. fluvialis* from *Vibrio cholerae*. Apart from the presence of bloody stool [41], *V. fluvialis* is almost indistinguishable from *V. cholerae* due to its similar clinical symptoms and colony morphology on Thiosulfate-Citrate-Bile-Sucrose agar [38]. In *V. fluvialis*, the ADI pathway comprises a cluster of *arcDACB* genes, whereas in *V. cholerae*, the absence of *arcC* and *arcD* leads to the inactivation of ADI pathway function [42]. The physiological function and regulatory mechanism of ADI in *V. fluvialis* are still unclear. In this study, the survival adaptation of *V. fluvialis* wild-type (WT) and isogenic ADI mutants in an acidic environment and the detailed regulatory mechanism of the ADI pathway by ArgR were investigated.

## 2. Results

### 2.1. Bioinformatic Analysis of ADI Gene Clusters and Regulatory Genes in the Genus Vibrio

**Figure 1:**
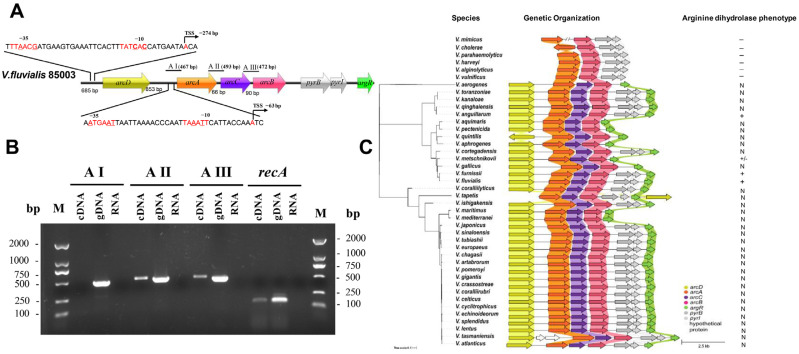
The genomic organizations of the *arc* gene cluster in *V. fluvialis* and in the genus *Vibrio*. (**A**) Genomic organization of ADI operons of *V. fluvialis*: *arcD*, arginine-ornithine antiporter; *arcA*, arginine deaminase; *arcC*, carbamate kinase; *arcB*, ornithine carbamoyltransferase; *argR*, arginine regulatory factor. The TSSs of *arcD* and *arcA* and the length of the spacer region between ORFs are shown above. The AI, AII, and AIII fragments with black lines of different lengths represent the *arc* gene sequences and the intergenic sequences amplified by PCR in panel (**B**). (**B**) RT-PCR analysis of the transcripts of the *arc* operon. Genomic DNA (gDNA) and unreversed RNA served as positive and negative controls, respectively. *recA:* internal reference. M: marker. (**C**) Composition and distribution of ADI gene clusters and their affinities in the genus *Vibrio*. The phylogenetic tree on the left shows the genetic relationships of the four proteins of the *arc* gene cluster in different *Vibrio* species. The tree scale represented a nucleotide substitution rate of 0.01 for each site. The genetic organization on the right shows the composition of the *arc* gene clusters, with different genes indicated by different colors. The length scale represents 2.5 kb. The presence of the arginine dihydrolase phenotype was represented as follows: “−”: negative; “+”, positive; “N”, unknown.

The ADI pathway in *V. fluvialis* appears to be clustered in an operon-like structure, which includes arginine deiminase (ArcA), ornithine carbamoyltransferase (ArcB), carbamate kinase (ArcC), and an arginine-ornithine antiporter (ArcD). Together with the regulatory protein ArgR, these proteins can functionally reverse the negative phenotype of arginine dihydrolase in *V. cholerae*, as we have demonstrated [42]. The ArcD, ArcA, ArcC, and ArcB coding genes are organized as an *arc* cluster approximately 5600 bp long, with noncoding regions 685 bp before *arcD*, 853 bp before *arcA*, 66 bp before *arcC*, and 90 bp before *arcB* (Figure 1A). The *argR* gene is located at a distant position downstream of *arcB* (Figure 1A). Reverse transcription (RT)-PCR using gene-specific and intergenic region-specific primers revealed that the *arc* cluster is organized into two operons, cotranscribed *arcACB* and separately transcribed *arcD* (Figure 1B). Subsequently, 5′-RACE was applied to determine the transcription start sites (TSSs) of the two operons. The TSSs were found to be located 274 bp upstream of the *arcD* start codon and 63 bp upstream of the *arcA* start codon (Figure 1A). Sequence analysis of the *arcD* promoter region revealed a putative −10 motif, TATCAC, and a −35 motif, TTAACG, with an 18 bp interval. Each putative motif has two mismatches (underlined bases) from the typical consensus motifs TATAAT and TTGACA. For the *arcA* promoter, sequence analysis predicted the elements TAAATT and ATGAAT as possible −10 and −35 motifs, which individually have two or three mismatches (underlined bases) with typical consensus sequences and are spaced at 15 bp intervals.

Then, we investigated the prevalence of the ADI gene cluster in the genus *Vibrio* using a tBLASTn search and compared its genetic organization. Although *arcA* and *arcB* homologs were identified in all *Vibrio* species with high similarity (>75%), no *arcC* or *arcD* homologs were found in *V. cholerae*, *V. parahaemolyticus*, *V. harveyi*, *V. alginolyticus*, or *V. vulnificus*, which all showed the species-specific negative phenotype of L-arginine utilization in the biochemical tests [43] (Figure 1C). A total of 36 known *Vibrio* species and 15 unknown *Vibrio* species in the NCBI database have complete *arcDACB* homologs, the amino acid sequences of which were mapped to the amino acid sequence of the *arc* cluster of *V. fluvialis* 33809. A phylogenetic tree based on the ArcDACB proteins from 36 species revealed that the *arc* gene cluster of *V. fluvialis* is closely related to those of *Vibrio furnissii* and *Vibrio gallicus* (Figure 1C). It is known that *V. fluvialis*, *V. furnissii*, and *V. anguillarum* have arginine dihydrolase-positive phenotypes [44,45,46]. Based on the high homology of ArcDACB proteins and similar genetic organization, we inferred that the other 33 known species may also exhibit an arginine dihydrolase-positive phenotype, which still needs biochemical confirmation. Together, our results showed the high conservation of *arc* gene clusters in different *Vibrio* species.

### 2.2. L-Arginine Enhances the Growth of V. fluvialis at Low pH

**Figure 2:**
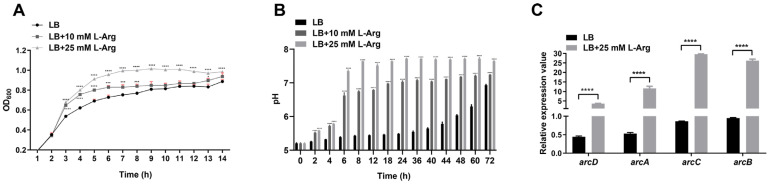
*V. fluvialis* responds to the presence of L-arginine during in vitro growth. (**A**,**B**) The *V. fluvialis* WT strain was grown in conventional LB (**A**) and acidic LB (pH = 5) (**B**) supplemented with 0, 10 mM, or 25 mM L-arginine, after which the OD_600_ of the culture (**A**) and the pH of the CS (**B**) were determined at indicated time points. (**C**) The WT strain was grown in LB supplemented with or without 25 mM L-arginine, and the transcription of the *arc* gene clusters was analyzed via qRT-PCR. The results are presented as the means ± SDs of three biological replicates, with the error bar highlighted in pink in (**A**). * *p*< 0.05, *** *p* < 0.001, and **** *p* < 0.0001.

Since L-arginine promotes the growth and expression of the ADI operon in other bacterial species [14,19,22,28], the role of L-arginine in *V. fluvialis* growth was investigated. As expected, exogenous L-arginine in LB medium greatly enhanced growth, especially in the late exponential growth phase of *V. fluvialis* (Figure 2A).

Because the conversion of arginine to ammonia by the ADI pathway could cause changes in culture pH, we next investigated the impact of L-arginine on pH in the culture supernatant (CS) of *V. fluvialis*. The WT was incubated at 37 °C in LB medium with an initial pH of 5. As shown in Figure 2B, the pH of CS increased in an arginine concentration-dependent pattern. The pH of L-arginine-supplemented CS increased rapidly at 4 h compared to that of the nonarginine control and increased to a greater value at 8 h, with the 10 mM group reaching nearly neutral and the 25 mM group even above a pH of 7.5. In contrast, the pH of the nonarginine control CS remained below 6 at 44 h and roughly reached neutral at 72 h. These data suggest that the ability of L-arginine to enhance the growth of *V. fluvialis* at low pH is correlated with the rapid increase in pH resulting from the production of ammonia from arginine by the ADI pathway. This finding was further supported by qRT-PCR analysis, which showed that the expression of the ADI gene cluster was strongly induced in response to the presence of arginine, with an 8-fold increase in the expression of *arcD*, a 20-fold increase in the expression of *arcA*, a 34-fold increase in the expression of *arcC* and a 28-fold increase in the expression of *arcB* (*p*-value < 0.0001) (Figure 2C).

### 2.3. Role of the ADI Gene Cluster and argR in Acid Resistance in V. fluvialis

**Figure 3:**
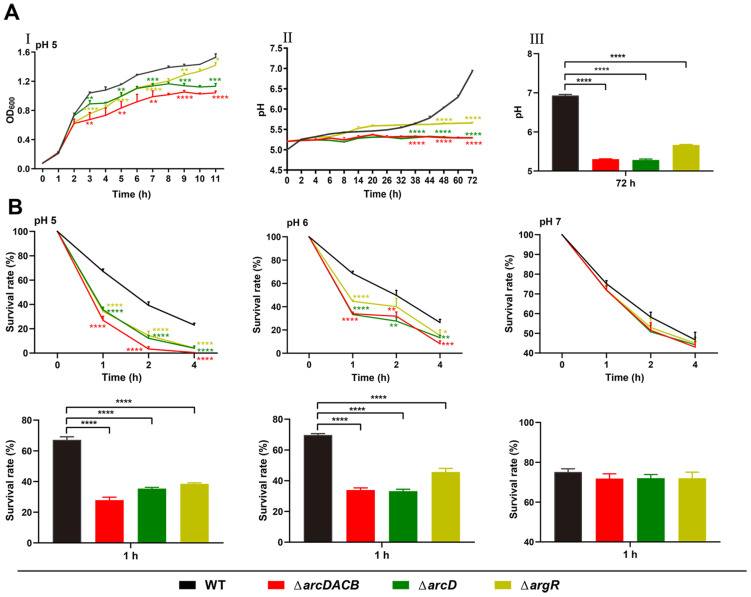
The effect of ADI and ArgR on the growth and survival of *V. fluvialis* under different pH environments. (**A**) I: Fresh overnight cultures of the WT and mutant strains were diluted (1:100) in fresh LB media at an initial pH of 5 and incubated at 37 °C for 12 h. The OD_600_ was measured every hour and the time points of 3, 5, 7, 9 and 11 h were used for the statistical analysis. II: Fresh overnight cultures of the WT and mutant strains were diluted (1:30) in fresh LB media at an initial pH of 5 and incubated at 37 °C for 72 h. The pH of the supernatant was measured for 72 h and the time points of 38, 48, and 72 h were used for statistical analysis. III: pH values of the WT and mutant strains at 72 h in II. (**B**) Acid resistance of the WT and mutant strains to different pH were monitored as described in the Materials and Methods Section. The results are presented as percentages of survival (CFU counts at corresponding time points compared to those in the initial inoculum). Line graphs from left to right show the survival rates of the WT strain and each mutant strain at different time points (1, 2, and 4 h) at pH 5, 6, and 7. The bar graphs below show the survival rates of the WT strain and each mutant strain at the corresponding pH at a 1 h time point. The results are presented as the means ± SDs of three biological replicates. * *p*< 0.05, ** *p* < 0.01, *** *p* < 0.001, and **** *p* < 0.0001.

To better understand the role of the ADI cluster in acid resistance, the in-frame deletion mutants of *arcDACB*, *arcD*, and *argR* were constructed. First, the WT, Δ*arcDACB*, Δ*arcD*, and Δ*argR* strains were cultured in acidic LB media (pH = 5) to explore their growth curves. As shown in Figure 3A(I), the Δ*arcDACB*, Δ*arcD*, and Δ*argR* strains all grew at lower rates than did the WT strain; Δ*arcDACB* grew at the slowest rate, followed by the Δ*arcD* and Δ*argR* strains which grew at roughly similar rates. Notably, there was no difference in their growth trends under neutral conditions (pH = 7) (Supplementary Figure S1A). Then, we monitored the pH of the CS of the WT strain and each mutant strain under acidic conditions (pH = 5) for 72 h (Figure 3A(I,III)). The pH of the WT CS slowly and gradually increased with incubation time and finally reached 7, while the pH of the CSs of Δ*argR*, Δ*arcDACB*, and Δ*arcD* remained below 5.5 during the whole incubation period, although the pH of the Δ*argR* exhibited a minor increase compared to that of the Δ*arcDACB* and Δ*arcD*, which may indicate that the ADI pathway still has some weak activity in the *argR*-negative background. These data indicate that the *argR*, *arcD*, and *arcACB* operons are individually necessary to maintain the function of the ADI pathway in *V. fluvialis*.

Subsequently, we investigated the acid resistance of the WT and each mutant strain in acidic solutions at pH 5 and 6, and the neutral condition (pH = 7) was used as the control. In general, all strains demonstrated high sensitivity to acidic conditions and showed a lower survival rate during longer incubation times. However, the survival rate of the WT strain was always notably greater than that of the *arc* mutants (Figure 3B). Additionally, the survival defects became more severe with decreased pH. At a pH of 5, much less survival of the Δ*arcDACB*, Δ*arcD*, and Δ*argR* was observed than that at a pH of 6, especially at both the 2 h and 4 h time points. Comparatively, under neutral conditions (pH = 7), the viabilities of the WT and the *acr* deletion mutants did not significantly differ, consistent with the similar growth curves presented in Supplementary Figure S1A. These findings corroborate that although not indispensable for growth, the ADI pathway and ArgR play a role in the acid resistance of *V. fluvialis*, and their absence significantly attenuates the survival of *V. fluvialis* under acidic conditions.

### 2.4. ArgR Activates arcD at the Transcriptional Level by Directly Binding to Its Promoter Region

**Figure 4:**
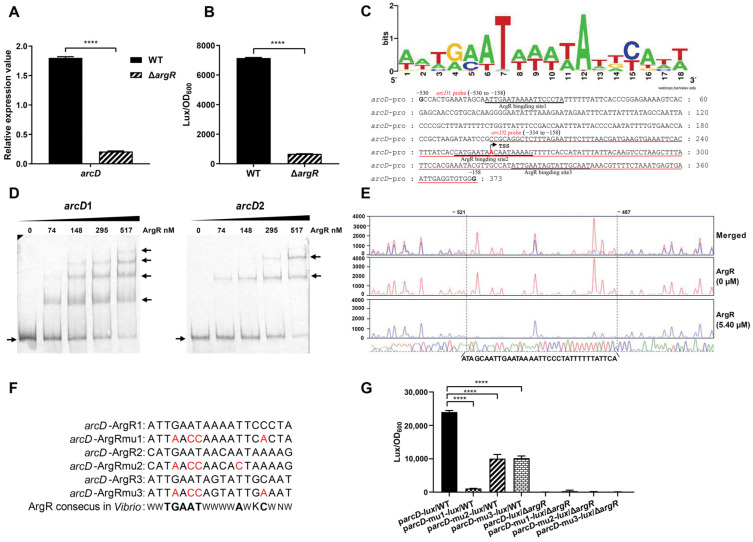
ArgR physically binds to the promoter region of *arcD*. (**A**) Determination of the mRNA level of *arcD* in the WT and Δ*argR* mutant strains. (**B**) Luminescence activity of p*arcD*-*lux* in the WT and Δ*argR* mutant strains. (**C**) The ArgR consensus sequence of *Vibrio* and the characterization of the *arcD* promoter region. The three potential ArgR binding sites are underlined, and the EMSA probe sequences (*arcD*1 probe: position −530 to −158; *arcD*2 probe: position −334 to −158) are marked and underlined in red. The TSS of *arcD* is indicated by an arrow and labeled with a red “A”. The numbers above the sequence indicate the positions of nucleotides relative to the *arcD* start codon. (**D**) The binding of ArgR to the promoter region of *arcD* is determined by EMSA. Biotin-labeled *arcD*1 (373 bp) or *arcD*2 (177 bp) DNA probes (15 ng for each) were incubated with purified ArgR-His_6_ protein. The arrows on the right indicated the shift bands of probes binding to the proteins, and the arrows on the left indicated the unbound probes. (**E**) DNase I footprinting assay of ArgR binding to the promoter region of *arcD*. The region protected by ArgR is marked with the box with a dotted line, and the nucleotide positions in the *arcD* promoter are labeled. The red and blue sequence traces represent different concentrations of ArgR (0 μM and 5.40 μM, respectively). The sequence traces of the four different colors representing the sequence of the *arcD* promoter are shown at the bottom. (**F**) The p*arcD*mu1-*lux*, p*arcD*mu2-*lux*, and p*arcD*mu3-*lux* were constructed by introducing 4-bp substitutions in the ArgR consensus site at the *arcD* promoter region. The red font represents the mutation site. (**G**) Luminescence activities of p*arcD*-lux, p*arcD*mu1-*lux*, p*arcD*mu2-*lux*, and p*arcD*mu3-*lux* in the WT and Δ*argR* strains. The luminescence activities are reported as light units/OD_600_. The results are presented as the means ± SDs of three biological replicates. **** *p* < 0.0001.

To determine whether ArgR regulates *arcD* expression, we examined the mRNA levels of *arcD* in the WT and Δ*argR* mutant strains. Figure 4A reveals a significant reduction in the *arcD* mRNA level in the Δ*argR* strain compared to that in the WT strain. To further elucidate the impact of ArgR on *arcD* promoter activity, the pBBR-*lux* vector carrying a promoterless *luxCDABE* reporter gene cluster was used to construct the fusion reporter plasmid p*arcD*-*lux*, which was transformed into the WT and Δ*argR* mutant strains. Compared to that of the WT strain, the luminescence activity of p*arcD*-*lux* was much lower in the Δ*argR* strain (Figure 4B), implying that ArgR activates *arcD* transcription.

ArgR is a regulator of the arginine pathway that binds to a conserved 14–20 bp refolding sequence named the ARG box in the target gene promoter region to regulate its expression [47]. The ArgR consensus in *Vibrio* comprises 18 nucleotides with relatively high AT content, and the sequence is as follows: 5′-WWTGMATWWWWATKCANW-3′ (where W = A or T, M = A or C, K = G or T, R = A or G, Y = T or C, N = any nucleotide) (https://regprecise.lbl.gov/sites.jsp?regulog_id=2347, accessed on 24 July 2023). To explore whether ArgR directly binds to the *arcD* promoter region, we initially analyzed the *arcD* promoter sequences for potential ARG boxes. Based on the conserved motif, three potential binding sites were identified at the −515 to −498, −282 to −265, and −209 to −192 positions relative to the *arcD* start codon (Figure 4C).

To validate the direct binding of ArgR to the predicted ArgR binding sites in the *arcD* promoter region, EMSA was performed with purified recombinant ArgR-His_6_ protein. We generated two biotin-labeled probes, namely, *arcD*1 (373 bp long with three potential binding sites) and *arcD*2 (177 bp long with two potential binding sites) (Figure 4C). Different amounts of ArgR-His_6_ proteins were incubated with 15 ng of the *arcD*1 or *arcD*2 probe. As shown in Figure 4D, four clearly shifted bands appeared for the *arcD*1 probe and two for the *arcD*2 probe, with the band intensity increasing with increasing ArgR-His_6_ concentration, indicating a direct interaction between the *arcD* probes and the recombinant ArgR-His_6_ proteins. For the *arcD* probe, the appearance of an extra band than expected may imply another potential binding site or non-specific binding at the highest protein concentration.

To further define the binding sequences of ArgR, we conducted a DNase I footprinting assay with a fluorescent FAM-labeled *arcD* probe containing the three predicted binding sites. DNase I footprinting assay revealed a nondigested ArgR-protected region comprising a 35 bp long sequence extending from the −521 to −486 positions relative to the *arcD* start codon (Figure 4E). This region encompasses the predicted ArgR binding site 1 (−515 to −498). Although the EMSA results for the *arcD*2 probe revealed two ArgR-shifted bands, indicating the presence of the predicted ArgR binding sites 2 and 3, these bands were not detected by DNase I footprinting analysis. This difference might be attributed to the lower affinity of ArgR for sites 2 and 3 than for site 1.

To further confirm and dissect the individual contributions of ArgR binding sites 1, 2, and 3 to *arcD* promoter activity, site mutations were performed in p*arcD*-*lux* to generate p*arcD*mu1-*lux*, p*arcD*mu2-*lux*, and p*arcD*mu3-*lux*. Each binding site underwent four-point mutation, substituting conserved G-AT with A-CC in all binding sites, the fourth-to-last C with A in sites 1 and 3, and the seventh-to-last A with C in site 2 (Figure 4F). These point-mutated recombinant plasmids were then transformed into the WT and Δ*argR* mutant strains to measure the luminescence. In the WT background, the luminescence of p*arcD*mu1-*lux* dramatically decreased by 22-fold compared to that of p*arcD*-*lux*, while the luminescence of p*arcD*mu2-*lux* and p*arcD*mu3-lux decreased 2-fold (*p* value < 0.0001). No significant difference in luminescence was observed for the Δ*argR* mutant (Figure 4G). Collectively, these results indicated that the introduced mutation in the ArgR binding sites disrupted the binding of ArgR, resulting in reduced luminescence activity. Furthermore, although all three predicted ArgR binding sites contribute to *arcD* transcriptional activation, the effect of ArgR binding site 1 is considerably greater than these of ArgR binding sites 2 and 3.

### 2.5. ArgR Activates the arcACB Operon by Physically Binding to the Promoter Region

**Figure 5:**
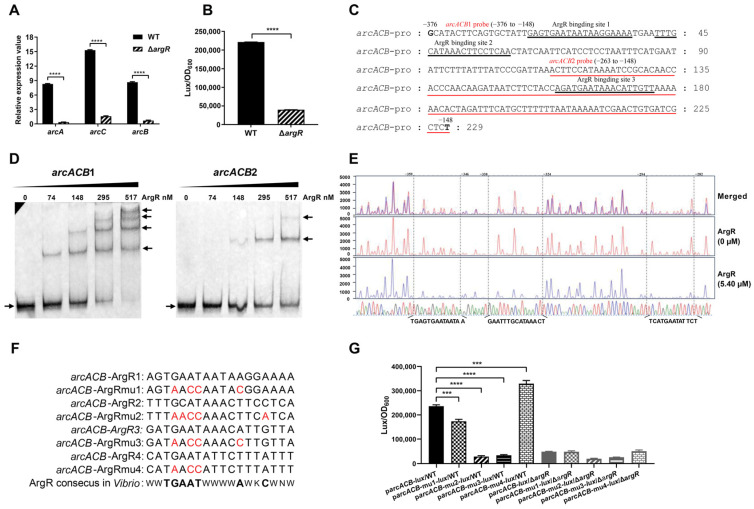
ArgR directly binds to the promoter region of *arcACB* operon. (**A**) Examination of the mRNA levels of *arcA*, *arcC*, and *arcB* in the WT and Δ*argR* mutant strains. (**B**) Luminescence activity of p*arcACB*-lux in the WT and Δ*argR* mutant strains. (**C**) Characterization of the *arcACB* operon promoter region. The four potential ArgR binding sites are underlined. The EMSA probe sequences (*arcACB1* probe: position −376 to −148; *arcACB2* probe: position −263 to −148) are marked and underlined in red. The numbers above the sequence indicate the positions of the nucleotides relative to the *arcA* start codon. (**D**) The binding of ArgR to the promoter region of *arcACB*, as determined by EMSA. The method and labeling were the same as those in Figure 4D. The arrows on the right of the gel pictures indicated the shift bands of probes binding to the proteins, and the arrows on the left of the gel pictures indicated the unbound probes. (**E**) The binding of ArgR to the promoter region of *arcACB* was determined by a DNase I footprinting assay. The method and labeling were the same as those in Figure 4E. The regions protected by ArgR are marked with three boxes with dotted line, and the nucleotide positions in the *arcACB* promoter are labeled. The red and blue sequence traces represent different concentrations of ArgR (0 μM and 5.40 μM, respectively). The sequence traces of four different colors representing the sequence of the *arcACB* promoter are shown at the bottom. (**F**) The p*arcACB*mu1-*lux*, p*arcACB*mu2-*lux*, p*arcACB*mu3-*lux* and p*arcACB*mu4-*lux* were constructed by introducing 4-bp changes in the ArgR consensus site at the *arcACB* promoter region. The red font represents the mutation site. (**G**) Luminescence activities of p*arcACB*-*lux*, p*arcACB*mu1-*lux*, parcACBmu2-lux, p*arcACB*mu3-*lux* and p*arcACB*mu4-*lux* in the WT and Δ*argR* strains. The luminescent activities were measured and are presented as light units/OD_600_. The results are presented as the means ± SDs of three biological replicates. *** *p* < 0.001, **** *p* < 0.0001.

To clarify whether ArgR regulates the *arcACB* operon, the mRNA levels of *arcA*, *arcC*, and *arcB* were examined in the WT and Δ*argR* mutant strains. As displayed in Figure 5A, the relative mRNA abundance of these genes was significantly lower in the Δ*argR* mutant than in the WT. To verify that the regulation occurred at the transcriptional level, a fusion reporter plasmid, p*arcACB*-*lux*, was constructed and introduced into the WT and Δ*argR* mutant. The luminescence activity of p*arcACB*-*lux* was notably lower in the Δ*argR* mutant than in the WT (Figure 5B). These findings suggest that ArgR plays a crucial role in activating the transcription of the *arcACB* gene cluster.

With the same strategy as *arcD*, the sequence of the promoter region of *arcACB* was analyzed in line with the ArgR consensus sequence in *Vibrio* (Figure 4C). Three potential ArgR binding sites were identified, ArgR binding site 1 (5′-AGTGAATAATAAGGAAAA-3′), binding site 2 (5′-TTTGCATAAACTTCC TCA-3′), and binding site 3 (5′-GATGAATAAACATTGTTA-3′), which are located at the −357 to −340, −335 to −318, and −217 to −200 positions, respectively, relative to the *arcA* start codon (Figure 5C). Next, we investigated the direct binding of ArgR to these binding sites using the EMSA and DNase I footprinting methods described above. EMSA revealed four shifted bands detected by the *arcACB*1 probes (229 bp, encompassing 3 predicted binding sites) and two shifted bands detected by the *arcACB*2 probes (116 bp, with the third binding site) (Figure 5D). DNase I footprinting analysis (Figure 5E) revealed three ArgR-protected regions located at positions −359 to −346 (5′-TGAGTGAATAATAA-3′), −338 to −324 (5′-GAATTTGCATAAACT-3′), and −294 to −282 (5′-TCATGAATATTCT-3′) relative to the *arcA* start codon. The percentages of the overlap between the first two protected regions and the predicted ArgR binding sites 1 and 2 were 86% and 80%, respectively. In contrast to EMSA, the DNase I footprinting method did not detect ArgR binding site 3 but detected an unpredicted new binding site, therefore, and we referred to ArgR binding site 4 below.

To functionally confirm these results, mutations were introduced into each predicted ArgR binding site in the *arcACB* promoter region using the same mutation strategy described above to generate p*arcACB*mu1-*lux*, p*arcACB*mu2-*lux*, p*arcACB*mu3-*lux*, and p*arcACB*mu4-*lux* based on p*arcACB*-*lux* (Figure 5F). These were then transformed into the WT and Δ*argR* mutant. The luminescence activities of p*arcACB*mu1-*lux*, p*arcACB*mu2-*lux* and p*arcACB*mu3-*lux* were 1.36-, 8.10-, and 6.83-fold lower than these of p*arcACB*-*lux* in the WT, while p*arcACB*mu4-lux showed enhanced activity (Figure 5G). In comparison, no significant differences were observed in the luminescence activities of these recombinant plasmids in the Δ*argR* mutant. The barely detectable luminescence activity of p*arcACB*mu3-*lux* in the WT, together with the EMSA results of the *arcACB*2 probe, suggested that the predicted ArgR binding site 3 is involved in the regulation of *arcACB* by ArgR. Additionally, p*arcACB*mu2-*lux* showed the lowest luminescence activity, suggesting that it may be the most functional ArgR binding site. The contrasting result of p*arcACB*mu4-*lux* is unexpected and needs to be investigated in the future.

### 2.6. Effects of the ADI Gene Cluster and argR on In Vivo Colonization of V. fluvialis

**Figure 6:**
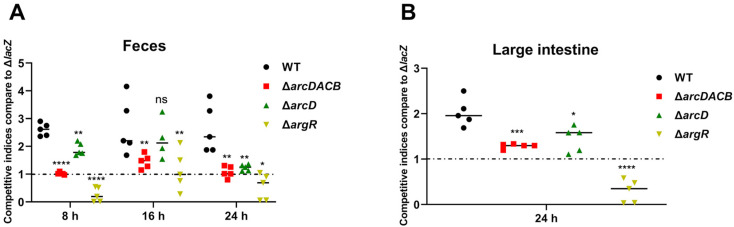
The deficiency of ArcD antiporter, the whole ADI or ArgR attenuates *V. fluvialis* colonization in mice. Female C57BL/6 mice (n = 5) were infected with equal amounts (1 × 10^8^ CFU each) of Δ*lacZ* and one of the competitive strains (WT, Δ*arcDACB*, Δ*arcD*, or Δ*argR*). (**A**) CI values of the WT, Δ*arcDACB*, Δ*arcD*, and Δ*argR* strains in the feces of the mice at 8 h, 16 h and 24 h postinfection; (**B**) CI values of the WT, Δ*arcDACB*, Δ*arcD* and Δ*argR* strains in the large intestine of the mice at 24 h postinfection after euthanasia. CI values above 1 indicate faster growth of the competitive strain than that of the Δ*lacZ* strain. * *p*< 0.05, ** *p* < 0.01, *** *p* < 0.001, **** *p* < 0.0001, and ns for no significant difference.

The acidic conditions of low pH in gastric fluid are often considered unfavorable for bacterial growth [48]. To further elucidate the role of the ADI pathway and its transcription factor ArgR in the in vivo infection of *V. fluvialis*, competitive colonization experiments were performed in mice to observe the colonization of *V. fluvialis*. Female C57BL/6 mice (*n* = 5) were infected by gavage with a mixture (1 × 10^8^ CFU each) of Δ*lacZ* and one of the competitive strains (WT, Δ*arcDACB*, Δ*arcD* or Δ*argR*), after which fecal and intestinal samples were collected for bacterial calculations as described in the Materials and Methods Section. As depicted in Figure 6, the trends for both the feces and large intestine results were generally consistent. Taking the 24 h results as an example, the CI values for the feces samples were 2.63 for WT, 1.08 for Δ*arcDACB*, 1.23 for Δ*arcD*, and 0.55 for Δ*argR*. For the large intestine samples, the CI values were 2.02 for WT, 1.30 for Δ*arcDACB*, 1.44 for Δ*arcD*, and 0.30 for Δ*argR*. Despite the similar growth abilities of the WT, Δ*arcDACB*, Δ*arcD*, and Δ*argR* strains in LB or M9 media (Supplementary Figure S1), deletion of either the ADI gene cluster or the *argR* gene severely attenuated the colonization of *V. fluvialis* in mice, suggesting a positive correlation between arginine metabolism and colonization.

### 2.7. ADI Pathway Deficiency Enhances the Phagocytosis of V. fluvialis by Macrophages

**Figure 7:**
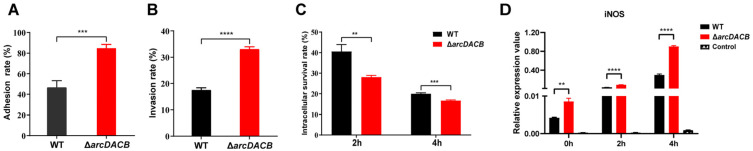
Role of the ADI in *V. fluvialis* phagocytosis by macrophages. Murine macrophages (RAW 264.7) were infected with WT or *arc*Δ*DACB* at an MOI of 8 using a gentamicin protection assay. The percentages of bacteria in adhesion (**A**), invasion (**B**), and intracellular survival (**C**) were calculated as the final CFU/mL postinfection divided by the original CFU/mL before infection. (**D**) The mRNA levels of iNOS in RAW 264.7 cells infected with WT or Δ*arcDACB*. Cell samples from the invasion (0 h), 2 and 4 h postinfection groups were collected for RNA extraction. qRT-PCR analysis is described in the Materials and Methods Section, with β-actin serving as an intracellular reference and uninfected RAW 264.7 cells serving as a control. The results are presented as the means ± SDs of three biological replicates. ** *p* < 0.01, *** *p* < 0.001, and **** *p* < 0.0001.

Since the ADI pathway can protect bacteria from lethal acidity in macrophages by increasing the cytoplasmic pH [49], we explored the effect of the ADI pathway on the survival of *V. fluvialis* in macrophages. WT and Δ*arcDACB* mutant strains were separately incubated with RAW 264.7 macrophages. Compared with the WT strain, the Δ*arcDACB* mutant strain exhibited enhanced macrophage adhesion and invasion, whereas intracellular survival was significantly reduced (Figure 7A–C).

NO is synthesized by nitric oxide synthase (iNOS) from arginine, and the bioavailability of arginine is one of the rate-limiting factors for intracellular NO production [50,51]. The ADI pathway utilizes intracellular arginine, creating competition. To further clarify the role of the ADI pathway in the infection of macrophages by *V. fluvialis*, we examined the mRNA levels of intracellular iNOS post infection. After 0 h of invasion into macrophages, the relative mRNA abundance of iNOS increased more in the Δ*arcDACB*-infected macrophage group than in the WT group at 2 h and 4 h post infection, with the increase being directly proportional to the incubation duration (Figure 7D). Taking the 4 h results as an example, the mRNA levels of iNOS were upregulated 106-fold in the Δ*arcDACB*-infected group, whereas they were upregulated 70-fold in the WT-infected group (Figure 7D). These preliminary results suggest that the ADI pathway might attenuate the phagocytic and immune responses of macrophages during *V. fluvialis* infection, thereby increasing the survival of *V. fluvialis* within macrophages.

## 3. Discussion

The ADI pathway, a prominent pathway for arginine catabolism in various microorganisms, has received extensive attention from researchers [14,17,25,27,28]. Bacteria expressing the ADI pathway can better adapt to hostile environments and evade host defenses, which has important implications for bacterial survival [28,29,52]. Specifically, the generation of ATP by the conversion of arginine to ornithine constitutes a major source of energy for bacteria during nutrient starvation. The NH_4_^+^ in the final product can change the pH in the environmental niche and enhance the fitness of bacteria. In addition, the intermediate metabolite ornithine contributes to the synthesis of polyamines, which are essential for tissue repair, anti-inflammatory responses [7], and also important for both the host (macrophages) and various intracellular pathogens like *Salmonella typhimurium* and *Mycobacterium tuberculosis*, etc. [6]. leading to investigations of new drag targets in the related pathways [53]. The ADI-positive (commonly called arginine dihydrolase-positive) phenotype is an important biochemical indicator for the identification of *V. fluvialis* species. In this study, we investigated the genetic organization, biological function, and regulatory mechanism of ADI in *V. fluvialis*, an emerging foodborne pathogen of public health concern. Our results showed that the deletion of the *arc* gene cluster significantly reduced the growth capacity of *V. fluvialis* under acidic conditions both in vitro (in culture medium and macrophages) and in vivo (in mice). Moreover, we revealed a positive regulatory mechanism of ArgR on *arcD* and *arcACB.*

The gene arrangement of the *arc* cluster differs among species [22], e.g., in *Streptococcus suis* [22], the arrangement was *arcABCD*, whereas in *S*. typhimurium [25], it was *arcACBD*; in *Pseudomonas aeruginosa* [54], it was *arcDABC*. In *V. fluvialis*, the *arc* gene cluster arrangement was *arcDACB*, and it was found to be highly conserved in the other *Vibrio species*, differing only in *Vibrio quintilis* and *Vibrio tapetis* (Figure 1C). Here, we showed that in *V. fluvialis*, the *arcDACB* gene cluster is organized into two transcriptional units. Specifically, the *arcD* is a monocistron, while the *arcA*, *arcC* and *arcB* are in one polycistron. 5′-Race revealed that *arcD* mRNA has a 274 bp long 5′ untranslated region (5′UTR) and that *arcACB* polycistronic mRNA has a short one with 63 bp long.

The comparison of the genetic content and organization of *arc* gene clusters in *V. fluvialis* and other *Vibrio* species revealed two major differences. In alignment with the biochemical arginine dihydrolase-negative phenotype, *V. mimicus*, *V. cholerae*, *V. parahaemolyticus*, *V. harveyi*, *V. alginolyticus*, and *V. vulnificus* lack the *arcD*, *arcC* and adjacent *argR* genes. The rest of the analyzed species contained the complete *arcD* gene, *arcACB* gene, and adjacent *argR* gene, of which the majority had the same genetic arrangement as *V. fluvialis*, *V. furnissii* and *V. anguillarum*, they are all arginine dihydrolase-positive phenotypes. Additionally, the *pyrB* and *pyrI* genes, which encode the aspartate carbamoyltransferase catalytic subunit and aspartate carbamoyltransferase regulatory chain, respectively, are associated with the core ADI cluster in *Vibrio* species without affecting the phenotype of arginine dihydrolase. Therefore, we reasoned that the presence of *arcD*, *arcACB*, and *argR* could be a preliminary indicator of the phenotype of positive arginine degradation in specific *Vibrio* species before experimental verification, and the genomic integrity could benefit the survival of *Vibrio* in nutrient-limited water niches.

It has been reported that *arc* catabolic gene expression is positively regulated by the regulator ArgR [20,32]. In *V. fluvialis*, ArgR also activates ADI expression through direct binding to the promoters of *arcD* and *arcACB* (Figure 4 and Figure 5). DNase I footprinting revealed one and three ArgR-protected DNA sequences in the *arcD* and *arcACB* promoter regions, respectively. These sequences were compared with the ArgR consensus sequences (ARG boxes) in *Vibrio* (Figure 4C) and other bacteria, such as *E. coli* [55], *Bacillus subtilis* [56], and *Streptococcus* [57]. These ArgR binding sites show highly conserved sequences of TGMAT but not strictly conserved palindromes, implying that the DNA-binding structural domains of ArgR may show different levels of conservation in various bacteria, but the DNA sequences they recognize remain relatively conserved.

ARG boxes typically achieve recognition specificity through cooperative interactions between tandem sites [58,59,60], and as such, these interactions usually occur in pairs, although single binding sites also exist [22,36,57,59]. The reported cooperation combined with the results of EMSA, DNase I footprinting, and point mutation (Figure 4D,E,G and Figure 5D,E,G), we also confirmed the presence of multiple ArgR binding sites on *arcD* and *arcACB*. We noticed that these binding sites show genomic position specificity. As for *arcD*, binding site 1 is the most critical one and is located far away from the TSS, while site 2 overlaps with the *arcD* TSS region, and site 3 is at the downstream of the TSS. Considering the long 5′UTR of *arcD* (−273 to the ATG start codon) and the locations of these binding sites, complex and fine modulation probably exist in *arcD* gene expression. As for *arcACB*, ArgR binding sites 2 and 3 were functionally confirmed to be the key cis-regulatory elements, which are all present upstream of the TSS of *arcA*. However, unlike site 2, EMSA confirmed site 3 but was not verified by DNase I footprinting analysis. This variation is probably due to the difference in the stability of the ArgR-DNA complex under the reaction conditions of the two assays but remains to be determined. Additionally, DNase I footprinting revealed an extra binding site 4 and was confirmed to inhibit *arcACB* expression by promoter-reporter fusion analysis, but the underlying mechanism and biological significance require more exploration in the following study. Considering that the *arcD* and *arcACB* promoters contain multiple ArgR binding sites with different affinities, whether the interactions between these sites increase the complexity of the regulatory mechanisms needs to be further investigated.

The ADI pathway, which is prevalent in various *Streptococcus* [22,28,61] and *P. aeruginosa* [14,54] strains, enables bacterial survival under lethal acidification by producing ammonia to increase the environmental pH [62]. Macrophage infection may further confirm this result, as the formation of phagocytic lysosomes in macrophages reduces the environmental pH to 4.4–4.7 [63]. Consistent with the literature, *V. fluvialis* WT exhibited faster pH regulation than the Δ*arcDACB*, Δ*arcD*, and Δ*argR* mutant strains under acidic conditions in vitro (Figure 3A), and this ability was further amplified by the utilization of L-arginine (Figure 2B). Deletion of the ADI pathway resulted in a significant reduction in acid survival (Figure 3B). During *V. fluvialis* infection of macrophages, the Δ*arcDACB* strain showed greater adhesion and invasion but lower intracellular survival than did the WT strain (Figure 7A–C). In addition to the lower acidic survival of Δ*arcDACB*, as we demonstrated above, we speculate that the lower intracellular survival is also related to the NO-mediated killing of macrophages. The ADI pathway competes for intracellular arginine; theoretically, Δ*arcDACB*-infected cells maintain higher intracellular arginine levels due to the loss of arginine catabolism. The infection of macrophages by the Δ*arcDACB* strain triggered a surge in intracellular iNOS expression (Figure 7D), which may enhance the throughput of intracellular iNOS to synthesize NO using arginine as a substrate and increase macrophage immunocompetence, leading to reduced survival of Δ*arcDACB*.

In vivo, competitive coinfection experiments in mice further demonstrated that the WT strain exhibited greater colonization ability in the feces and large intestine than did the mutant strains (Figure 6). This may be due to the greater acid resistance of the WT strain when it passed through the acidic environment of the stomach. An acid resistance assay also confirmed the high survival rate of the WT strain at a pH of 5 at 1 h, which was approximately three times greater than that of the Δ*arcDACB* strain (Figure 3B). The Δ*arcDACB* and Δ*arcD* strains exhibited similar growth rates in acidic environments and in a mouse competition assay, albeit significantly lower than that of the WT strain (Figure 3). This finding suggested a critical role of the transporter protein ArcD in *V. fluvialis* growth under acidic conditions, consistent with its role in maintaining cellular homeostasis and survival adaptation in *S. pneumoniae* [18] and *S. suis* [64]. However, the growth of the Δ*argR* strain in vivo differed from that in vitro. ArgR, as a global regulator, not only regulates the ADI pathway but also regulates other functional pathways, such as alcohol dehydrogenase and the ABC transporter system [18]. The weaker colonization of the Δ*argR* strains in vivo may be related to its broad regulatory capacity, which needs to be further investigated.

In conclusion, our study revealed that the ADI pathway significantly enhanced the growth capacity of *V. fluvialis* in acidic culture environments, in macrophages, and in the mouse large intestine, proving that the transcription factor ArgR is involved in the regulation of ADI through direct binding to the promoter regions of *arcD* and *arcACB*. These findings greatly enrich our understanding of arginine metabolism and regulation in *V. fluvialis*, providing a crucial basis for future research on the mechanisms of pathogenicity and environmental adaptation of *V. fluvialis*.

## 4. Materials and Methods

### 4.1. Bacterial Strains, Culture Conditions, and Plasmids

All bacterial strains and plasmids used in this study are listed in Supplementary Table S1, and the primers used are listed in Supplementary Table S2. The *V. fluvialis* 85003, isolated in 1985 from an adult patient with diarrhea in Xinjiang Province, China [42], was used as the WT strain, and the mutant strains Δ*arcDACB*, Δ*arcD*, and Δ*argR* were generated in this study. *E. coli* Top10 and SM10*λpir* were used for cloning and conjugation, respectively, and Rosetta (DE3) was used as the host for the expression and purification of ArgR-His_6_ which was amplified by PCR and inserted into the pET30a vector. All strains were grown in Luria–Bertani (LB) broth (Oxoid, Basingstoke, UK) containing 1% NaCl at 37 °C. For growth experiments, a 2.5 M L-arginine master mixture was added to the growth media at the indicated concentrations. Antibiotics were used at the following final concentrations (wt/vol) if necessary: ampicillin (Amp, 100 µg/mL); chloramphenicol (Cm, 10 µg/mL for *E. coli*, 3 µg/mL for *V. fluvialis*); streptomycin (Sm, 100 µg/mL); kanamycin (Kan, 50 μg/mL); isopropyl-b-D-thiogalac-topyranoside (IPTG) at a final concentration of 0.4 mM.

### 4.2. Distribution and Phylogenetic Analysis of the ADI Cluster in Vibrio Species

The amino acid sequences of the arginine deiminase ArcA (*arcA*, AMF95908), ornithine carbamoyltransferase ArcB (*arcB*, AMF95906), carbamate kinase ArcC (*arcC*, AMF95907), arginine-ornithine antiporter ArcD (*arcD*, AMF95909), and regulatory protein ArgR (*argR*, AMF95903) were retrieved from the NCBI database (NZ_CP014035.2). The homology of the five proteins in the NCBI nucleotide database (26–28 February 2023) was compared using a protein alignment-based nucleotide search method (tBLASTn, https://blast.ncbi.nlm.nih.gov/Blast.cgi, accessed on 26–28 February 2023), with a minimum of 60% coverage and 40% identity as the retrieval criteria for reference genome selection for different *Vibrio* species. Sequence information for the *arc* gene cluster was extracted from 42 different *Vibrio species*, and the specific *Vibrio* strain names are shown in Supplementary Table S3. The *arc* gene clusters were then concatenated to construct a neighbor-joining phylogenetic tree via MEGA7 [65]. iTOL (https://itol.embl.de/, accessed on 28 February 2023) was used for visualization, and clinker (https://github.com/gamcil/clinker, accessed on 28 February 2023) for gene cluster comparison.

### 4.3. Construction of in-Frame Deletion Mutants

In-frame deletion mutants of the WT strains Δ*arcDACB*, Δ*arcD*, and Δ*argR* were generated by allelic exchange. Briefly, approximately 1000 bp of the upstream and downstream flanking fragments of the targeting ORFs were amplified using the corresponding primers (Supplementary Table S2) and stitched together by an overlapping PCR method as described previously [66]. The 1779 bp Δ*arcDACB*, 1736 bp Δ*arcD*, and 1998 bp Δ*argR* fragments were cloned into the pWM91 suicide plasmid at the *Xba*I-*Sac*I sites, resulting in pWM91Δ*arcDACB*, pWM91Δ*arcD*, and pWM91Δ*argR*, respectively. Then, the recombinant suicide plasmids were introduced into *V. fluvialis* WT (recipient) strain from *E. coli* SM10*λpir* (donor) by conjugation. The transformants were screened in LB media supplemented with Amp and Sm and then counterselected by streaking on NaCl-free and sucrose-containing (10%) LB agar plates. Sucrose-resistant and Amp-sensitive strains were verified by PCR and confirmed by DNA sequencing.

### 4.4. RNA Extraction and Quantitative Real-Time PCR (qRT-PCR)

The WT and its derivative mutants were harvested at the late-logarithmic phase (OD_600_ ~1.0) for total RNA extraction, and cDNA synthesis was subsequently performed as previously described [66]. Three biological replicates were conducted for each sample. Nonreverse transcription RNA served as a negative control, *recA* served as a reference gene for *V. fluvialis* and β-actin served as a reference gene for RAW 264.7 cells. The relative expression values (R) were calculated using the equation *R* = 2^−(ΔCT target−ΔCT reference)^. The relevant primers used are listed in Supplementary Table S2, with iNOS and β-actin primers from Xu YW et al. [67].

### 4.5. 5′-Rapid Amplification of cDNA End (5′-RACE)

5′-RACE was performed using a SMARTer^®^ RACE 5′/3′ kit (Takara Bio Inc., Kusatsu, Japan). Total RNA extraction was the same as that for qRT-PCR. Following the manufacturer’s guidelines, 5′-RACE-ready cDNA samples were generated utilizing a random primer mixture. Then, 5′-RACE PCR was performed using a universal primer (UPM) and a gene-specific primer (GSP). The resulting PCR amplicon was then ligated into the pMD20 TA cloning vector and transformed into the Top 10 competent cells. Subsequently, the integrity of the inserted sequence was determined by DNA sequencing.

### 4.6. Growth Analysis

Growth curves were examined by microtiter plates as follows: overnight cultures of *V. fluvialis* strains were washed once in 1 volume of phosphate-buffered saline (PBS) and then diluted (1:100) into fresh LB medium with different L-arginine concentrations. Triplicates of 200 µL of diluted cultures were transferred to a 100-well microtiter plate and incubated at 37 °C with constant shaking at 200 rpm. The OD_600_ was measured every hour using a Bioscreen (Oy Growth Curve, Helsinki, Finland). The OD_600_ values at each time point were averaged and plotted against time to generate growth curves.

### 4.7. Acid Resistance of V. fluvialis Strains

The growth of *V. fluvialis* strains in low-pH LB was performed as follows: fresh overnight cultures of WT and its derivative mutants were diluted (1:30) in 40 mL of LB (pH 5). At different intervals up to 72 h, 2 mL of supernatant from each strain was collected for pH measurement. The survival of *V. fluvialis* was examined as described previously [22]. Briefly, 100 µL of fresh bacterial culture (1.5 × 10^8^ CFU/mL) was inoculated in an acidic solution (20 mM Na_2_HPO_4_, 1 mM MgCl_2_, 25 mM arginine-HCl) with a pH of 5, 6, or 7 and incubated at 37 °C for 1 h, 2 h, or 4 h. The surviving bacteria were quantified by the plate count method. Survival rates are presented as the ratios of surviving bacteria to input bacteria.

### 4.8. Analysis of the Transcriptional Activity of Promoters with a Luciferase Reporter Gene Assay

The promoter regions of *arcD* and *arcACB* were amplified and inserted into the upstream of the promoterless *luxCDABE* operon in pBBR*lux*. The resultant *lux* reporter fusion plasmids p*arcD-lux* and p*arcACB-lux* were introduced into the WT and ∆*argR* strains by conjugation from SM10*λpir*. Site mutations at the predicted ArgR binding sites were generated by PCR-based site-directed mutagenesis using the corresponding template. For example, p*arcD-lux* was used for p*arcD*mu1-*lux*, p*arcD*mu2-*lux*, and p*arcD*mu3-*lux*, while p*arcACB*-*lux* was used for p*arcACB*mu1-*lux*, p*arcACB*mu2-*lux*, p*arcACB*mu3-*lux*, and p*arcACB*mu4-*lux*. Overnight cultures of *V. fluvialis* strains harboring *lux* reporter fusion plasmids were diluted (1:100) in fresh LB media. Triplicates of 200 µL of diluted cultures were transferred to opaque 96-well microtiter plates and incubated at 37 °C with shaking at 200 rpm. Luminescence and OD_600_ were measured every hour using a spectrophotometer (Infinite M200 Pro, Tecan, Grödig, Austria). Luciferase activity was calculated as previously described [68].

### 4.9. Cloning, Expression, and Purification of ArgR-His_6_

The coding region of ArgR was PCR amplified and inserted into pET30a at the *Nde*I*/Xho*I sites. The resulting pET*argR* was subsequently transformed into Rosetta (DE3) for ArgR-His overexpression. The *E. coli* strain was cultured in LB with Kana at 37 °C with shaking at 200 rpm before being induced by 0.4 mM IPTG when the OD_600_ reached 0.4–0.6. Then, the culture was incubated at 16 °C for 20 h with shaking at 100 rpm. The ArgR-His_6_ protein was purified using affinity chromatography Ni^2+^ resin (Thermo Fisher Scientific, Waltham, MA, USA) and concentrated with an Ultra10 centrifugal filter (Merck, Darmstadt, Germany) according to the manufacturer’s instructions.

### 4.10. Electrophoretic Mobility Shift Assay (EMSA)

The promoter regions of *arcD* and *arcACB* were amplified with biotin-labeled primer pairs (Supplementary Table S2) using the p*arcD*-*lux* and p*arcACB*-*lux* plasmids, as templates, respectively. A 20 μL reaction mixture of 15 ng of biotin-labeled amplicons with increasing amounts of purified ArgR-His_6_ protein in binding buffer (20 mM Tris/HCl [pH 7.6], 50 mM KCl, 1 mM EDTA, 5% [vol/vol] glycerol) containing 200 ng of BSA and 500 ng of dI-dC was incubated at 30 °C for 20 min, separated in a native 8% polyacrylamide gel, transferred to a nylon membrane and visualized by a Chemiluminescent Nucleic Acid Detection Module (Thermo Fisher Scientific, Waltham, MA, USA).

### 4.11. DNase I Footprinting Assay

DNase I footprinting and sequencing assays were performed as previously described [69]. Briefly, the promoter regions of *arcD* and *arcACB* of the WT were amplified with a high-fidelity PCR kit (Sangon Biotech, Shanghai, China) using the primer pairs p*arcD*-F (FAM)/p*arcD*-R and p*arcACB*-F (FAM)/p*arcACB*-R (Supplementary Table S2) to generate the *arcD* and *arcACB* probes, respectively. The probes, 371 ng of *arcACB* and 468 ng of *arcD*, were mixed with 5.40 μM ArgR-His_6_ in a 40 μL final reaction volume. After incubation at 37 °C for 0.5 h, the reaction mixture was digested with 0.015 units of RNase-Free DNase I (Promega, Madison, WI, USA), sequenced with an Applied Biosystems 3500XL DNA Analyzer (Thermo Fisher Scientific, Waltham, MA, USA) and analyzed with Peak Scanner software v1.0 (Thermo Fisher Scientific, Waltham, MA, USA).

### 4.12. Mouse Competition Assay

The competition assay was performed based on the principle that the color of colonies on X-gal chromogenic plates changes from blue to white for the *V. fluvialis* 85003 *lacZ* deletion mutant. Overnight cultures of the WT, Δ*arcDACB*, Δ*arcD*, Δ*argR*, and Δ*lacZ* strains were diluted (1:100) in fresh LB media and incubated for 3 h at 37 °C until an OD_600_ of 0.3 was reached. The bacterial suspensions were centrifuged at 5000 rpm for 3 min before the pellets were resuspended in PBS. An equal volume of bacteria (1 × 10^9^ CFU/mL for each) was mixed, serially diluted, and spread on LB agar plates supplemented with Sm and X-gal for live bacteria plate counting. The input ratios of each strain to Δ*lacZ* were calculated and adjusted to a ratio of 1:1 for mouse infection. Groups of five Sm-pretreated six-week-old female C57BL/6 mice (Beijing Vital River Laboratory Animal Technology Co., Ltd., Beijing, China) were infected with 100 µL of the mixed suspension (1 × 10^8^ CFU for each) by intragastric gavage. After 24 h, all mice underwent cervical dislocation following carbon dioxide euthanasia. Output ratios of bacteria in feces and large intestines (colon and cecum) were determined from homogenated feces at 8 h, 16 h, and 24 h or tissues at 24 h postinfection by serial dilution plate counting for live bacteria. The competitive index (CI) is defined as the output ratio divided by the input ratio, with CI values less than 0.5 considered significant.

All experiments involving animals were performed according to protocols approved by the Animal Care and Use Committee of the National Institute for Communicable Disease Control and Prevention and according to the medical research regulations of the National Health Commission, China (protocol number 2023-014).

### 4.13. Adhesion, Invasion, and Intracellular Survival of V. fluvialis in RAW 264.7 Cells

Murine macrophage RAW 264.7 cells were cultured in high glucose (4.5 g/L) Dulbecco’s modified Eagle’s medium (DMEM) supplemented with 10% heat-inactivated fetal bovine serum (Gibco, Grand Island, NY, USA) at 37 °C with 5% CO_2_. The gentamicin protection assay was used for invasion and intracellular survival tests [70]. For this aim, RAW 264.7 cells (2 × 10^5^ CFU/well) were seeded into 24-well plates and incubated in an antibiotic-free medium. For the adhesion assay, fresh bacteria at an OD_600_ of 0.3 were prepared as described previously, and these bacteria were serially diluted and counted on LB plates to serve as the original pre-infection bacterial count (CFU/mL). Then, the bacteria were washed with PBS and resuspended in DMEM for RAW 264.7 cell infection at a multiplicity of infection (MOI) of 1:8 and seeded into a 24-well plate. The plate was centrifuged at 1000× *g* for 5 min and incubated at 37 °C for 1 h. Then, the infected cells were washed three times with PBS to remove unattached bacteria and lysed with lysis buffer (0.1% SDS and 1% Triton X-100 in PBS). The lysate was then serially diluted and spread on LB agar plates. The plates were incubated at 37 °C overnight for live bacteria plate counting (CFU/mL). For invasion, RAW 264.7 cells were infected as described for the adhesion assay, except for treating with gentamicin (500 μg/mL) for 1 h to kill extracellular bacteria immediately after the PBS wash step. For intracellular survival, after gentamicin treatment, the infected cells were washed with PBS and incubated for an additional 4 h in fresh DMEM containing 10 μg/mL gentamicin. Survival rates are presented as the ratios of the CFU in the intracellular survival assay to the CFU in the invasion assay.

### 4.14. Statistical Analysis

GraphPad Prism software, version 8, was used for statistical analysis. Statistical significance was determined by unpaired two-tailed Student’s *t*-test and was defined as * if the *p*-value was less than 0.05, ** if the *p*-value was less than 0.01, *** if the *p-*value was less than 0.001, **** if the *p*-value was less than 0.0001, and ns for no significant difference.

## Data Availability

No datasets were generated or analyzed during the current study. The original contributions presented in the study are included in the article/Supplementary Materials, further inquiries can be directed to the corresponding authors.

## Supplementary Materials

The supporting information can be downloaded at: https://www.mdpi.com/article/10.3390/ijms25115679/s1.

## References

[1] Hornef M.W., Wick M.J., Rhen M., Normark S. (2002). Bacterial strategies for overcoming host innate and adaptive immune responses. Nat. Immunol..

[2] Tlaskalová-Hogenová H., Štěpánková R., Hudcovic T., Tučková L., Cukrowska B., Lodinová-Žádníková R., Kozáková H., Rossmann P., Bártová J., Sokol D. (2004). Commensal bacteria (normal microflora), mucosal immunity and chronic inflammatory and autoimmune diseases. Immunol. Lett..

[3] Donnenberg M.S., Whittam T.S. (2001). Pathogenesis and evolution of virulence in enteropathogenic and enterohemorrhagic *Escherichia coli*. J. Clin. Investig..

[4] Hooper L.V., Gordon J.I. (2001). Commensal Host-Bacterial Relationships in the Gut. Science.

[5] Lynch J.P., Lesser C.F. (2021). A host metabolite promotes *Salmonella* survival. Science.

[6] Manchester M., Das P., Lahiri A., Lahiri A., Chakravortty D. (2010). Modulation of the Arginase Pathway in the Context of Microbial Pathogenesis: A Metabolic Enzyme Moonlighting as an Immune Modulator. PLoS Pathog..

[7] Gogoi M., Datey A., Wilson K.T., Chakravortty D. (2016). Dual role of arginine metabolism in establishing pathogenesis. Curr. Opin. Microbiol..

[8] Abdelal A.T. (1979). Arginine Catabolism by Microorganisms. Annu. Rev. Microbiol..

[9] Stadelmann B., Hanevik K., Andersson M.K., Bruserud O., Svärd S.G. (2013). The role of arginine and arginine-metabolizing enzymes during Giardia—Host cell interactions in vitro. BMC Microbiol..

[10] Xiong L.F., Teng J.L.L., Botelho M.G., Lo R.C., Lau S.K.P., Woo P.C.Y. (2016). Arginine Metabolism in Bacterial Pathogenesis and Cancer Therapy. Int. J. Mol. Sci..

[11] Hernández V.M., Arteaga A., Dunn M.F. (2021). Diversity, properties and functions of bacterial arginases. Fems Microbiol. Rev..

[12] Xiong L., Teng J.L.L., Watt R.M., Kan B., Lau S.K.P., Woo P.C.Y. (2014). Arginine deiminase pathway is far more important than urease for acid resistance and intracellular survival in *Laribacter hongkongensis*: A possible result of arc gene cassette duplication. BMC Microbiol..

[13] Weathers P.J., Chee H.L., Allen M.M. (1978). Arginine catabolism in *Aphanocapsa* 6308. Arch. Microbiol..

[14] Lu C.-D., Winteler H., Abdelal A., Haas D. (1999). The ArgR Regulatory Protein, a Helper to the Anaerobic Regulator ANR during Transcriptional Activation of the *arcD* Promoter in *Pseudomonas aeruginosa*. J. Bacteriol..

[15] Minic Z., Hervé G. (2003). Arginine Metabolism in the Deep Sea Tube Worm *Riftia pachyptila* and Its Bacterial Endosymbiont. J. Biol. Chem..

[16] Caldara M., Charlier D., Cunin R. (2006). The arginine regulon of *Escherichia coli*: Whole-system transcriptome analysis discovers new genes and provides an integrated view of arginine regulation. Microbiology.

[17] Liu Y., Dong Y., Chen Y.-Y.M., Burne R.A. (2008). Environmental and Growth Phase Regulation of the *Streptococcus gordonii* Arginine Deiminase Genes. Appl. Environ. Microbiol..

[18] Schulz C., Gierok P., Petruschka L., Lalk M., Mäder U., Hammerschmidt S., Thornton J.A., McDaniel L.S. (2014). Regulation of the Arginine Deiminase System by ArgR2 Interferes with Arginine Metabolism and Fitness of *Streptococcus pneumoniae*. mBio.

[19] Coburn J., Richards C.L., Raffel S.J., Bontemps-Gallo S., Dulebohn D.P., Herbert T.C., Gherardini F.C. (2022). The arginine deaminase system plays distinct roles in *Borrelia burgdorferi* and *Borrelia hermsii*. PLOS Pathog..

[20] Ryan S., Begley M., Gahan C.G.M., Hill C. (2009). Molecular characterization of the arginine deiminase system in *Listeria monocytogenes*: Regulation and role in acid tolerance. Environ. Microbiol..

[21] Lindgren J.K., Thomas V.C., Olson M.E., Chaudhari S.S., Nuxoll A.S., Schaeffer C.R., Lindgren K.E., Jones J., Zimmerman M.C., Dunman P.M. (2014). Arginine Deiminase in *Staphylococcus epidermidis* Functions to Augment Biofilm Maturation through pH Homeostasis. J. Bacteriol..

[22] Gruening P., Fulde M., Valentin-Weigand P., Goethe R. (2006). Structure, Regulation, and Putative Function of the Arginine Deiminase System of *Streptococcus suis*. J. Bacteriol..

[23] Xu B., Yang X.Y., Zhang P., Ma Z., Lin H.X., Fan H.J. (2016). The arginine deiminase system facilitates environmental adaptability of *Streptococcus equi* ssp. zooepidemicus through pH adjustment. Res. Microbiol..

[24] Cotter P.D., Gahan C.G.M., Hill C. (2000). Analysis of the role of the *Listeria* monocytogenes F0F1-ATPase operon in the acid tolerance response. Int. J. Food Microbiol..

[25] Choi Y., Choi J., Groisman E.A., Kang D.-H., Shin D., Ryu S., Camilli A. (2012). Expression of STM4467-Encoded Arginine Deiminase Controlled by the STM4463 Regulator Contributes to *Salmonella enterica* Serovar Typhimurium Virulence. Infect. Immun..

[26] Chakraborty B., Burne R.A. (2017). Effects of Arginine on *Streptococcus* mutans Growth, Virulence Gene Expression, and Stress Tolerance. Appl. Environ. Microbiol..

[27] Makhlin J., Kofman T., Borovok I., Kohler C., Engelmann S., Cohen G., Aharonowitz Y. (2007). *Staphylococcus aureus* ArcR controls expression of the arginine deiminase operon. J. Bacteriol..

[28] Hitzmann A., Bergmann S., Rohde M., Chhatwal G.S., Fulde M. (2013). Identification and characterization of the arginine deiminase system of *Streptococcus canis*. Vet. Microbiol..

[29] Chantratita N., Tandhavanant S., Wikraiphat C., Trunck L.A., Rholl D.A., Thanwisai A., Saiprom N., Limmathurotsakul D., Korbsrisate S., Day N.P.J. (2012). Proteomic analysis of colony morphology variants of *Burkholderia pseudomallei* defines a role for the arginine deiminase system in bacterial survival. J. Proteom..

[30] Maghnouj A., Abu-Bakr A.A.W., Baumberg S., Stalon V., Wauven C. (2000). Regulation of anaerobic arginine catabolism in *Bacillus licheniformis* by a protein of the Crp/Fnr family. FEMS Microbiol. Lett..

[31] Winterhoff N., Goethe R., Gruening P., Rohde M., Kalisz H., Smith H.E., Valentin-Weigand P. (2002). Identification and Characterization of Two Temperature-Induced Surface-Associated Proteins of *Streptococcus suis* with High Homologies to Members of the Arginine Deiminase System of *Streptococcus pyogenes*. J. Bacteriol..

[32] Fulde M., Willenborg J., de Greeff A., Benga L., Smith H.E., Valentin-Weigand P., Goethe R. (2011). ArgR is an essential local transcriptional regulator of the *arcABC* operon in *Streptococcus suis* and is crucial for biological fitness in an acidic environment. Microbiology.

[33] Grandori R., Lavoie T.A., Pflumm M., Tian G., Niersbach H., Maas W.K., Fairman R., Carey J. (1995). The DNA-binding Domain of the Hexameric Arginine Repressor. J. Mol. Biol..

[34] Garnett J.A., Marincs F., Baumberg S., Stockley P.G., Phillips S.E.V. (2008). Structure and Function of the Arginine Repressor-Operator Complex from *Bacillus subtilis*. J. Mol. Biol..

[35] Cherney L.T., Cherney M.M., Garen C.R., James M.N.G. (2010). Crystal Structure of the Intermediate Complex of the Arginine Repressor from *Mycobacterium tuberculosis* Bound with Its DNA Operator Reveals Detailed Mechanism of Arginine Repression. J. Mol. Biol..

[36] Maghnouj A., de Sousa Cabral T.F., Stalon V., Vander Wauven C. (1998). The *arcABDC* Gene Cluster, Encoding the Arginine Deiminase Pathway of *Bacillus licheniformis*, and Its Activation by the Arginine Repressor ArgR. J. Bacteriol..

[37] Chatterjee B., Thawani G., Sanyal S. (1989). Etiology of acute childhood diarrhoea in Calcutta. Trop Gastroenterol.

[38] Chowdhury G., Pazhani G.P., Dutta D., Guin S., Dutta S., Ghosh S., Izumiya H., Asakura M., Yamasaki S., Takeda Y. (2012). *Vibrio fluvialisin* Patients with Diarrhea, Kolkata, India. Emerg. Infect. Dis..

[39] Kobayashi K., Taguchi M., Shimada T., Sakazaki R. (1983). Ten cases of gastroenteritis possibly caused by *Vibrio fluvialis* and its enterotoxigenicity. J. Jpn. Assoc. Infect. Dis..

[40] Igbinosa E.O., Obi L.C., Okoh A.I. (2009). Occurrence of potentially pathogenic *Vibrios* in final effluents of a wastewater treatment facility in a rural community of the Eastern Cape Province of South Africa. Res. Microbiol..

[41] Huq M.I., Alam A.K., Brenner D.J., Morris G.K. (1980). Isolation of *Vibrio*-like group, EF-6, from patients with diarrhea. J. Clin. Microbiol..

[42] Lu X., Liang W., Wang Y., Xu J., Zhu J., Kan B. (2014). Identification of Genetic Bases of *Vibrio fluvialis* Species-Specific Biochemical Pathways and Potential Virulence Factors by Comparative Genomic Analysis. Appl. Environ. Microbiol..

[43] Xiao D., Yang W., Wang H., Bi Z., Lu L., Kan B., Wang L., Wang D., Wang M., Wang G., Feng Z., Bi Z., Su H., Yang W., Xiao D., Wang H. (2013). Vibrio Cholera Prevention and Control Manual.

[44] Brenner D.J., Hickman-Brenner F.W., Lee J.V., Steigerwalt A.G., Fanning G.R., Hollis D.G., Farmer J.J., Weaver R.E., Joseph S.W., Seidler R.J. (1983). *Vibrio furnissii* (formerly aerogenic biogroup of *Vibrio fluvialis*), a new species isolated from human feces and the environment. J. Clin. Microbiol..

[45] Liang P., Cui X., Du X., Kan B., Liang W. (2013). The virulence phenotypes and molecular epidemiological characteristics of *Vibrio fluvialis* in China. Gut Pathog..

[46] Kala K., Shahi N., Singh S., Rawat S., Patiyal R.S., Pande V., Mallik S.K. (2021). New host record of *Vibrio anguillarum* associated with haemorrhagic septicaemia in golden mahseer, Tor putitora (Hamilton, 1822) from India. Indian J. Comp. Microbiol. Immunol. Infect. Dis..

[47] Makarova K.S., Mironov A.A., Gelfand M.S. (2001). Conservation of the binding site for the arginine repressor in all bacterial lineages. Genome Biol..

[48] Franzosa E.A., Morgan X.C., Segata N., Waldron L., Reyes J., Earl A.M., Giannoukos G., Boylan M.R., Ciulla D., Gevers D. (2014). Relating the metatranscriptome and metagenome of the human gut. Proc. Natl. Acad. Sci. USA.

[49] Degnan B.A., Tuomanen E.I., Fontaine M.C., Doebereiner A.H., Lee J.J., Mastroeni P., Dougan G., Goodacre J.A., Kehoe M.A. (2000). Characterization of an Isogenic Mutant of *Streptococcus pyogenes* Manfredo Lacking the Ability To Make Streptococcal Acid Glycoprotein. Infect. Immun..

[50] Alderton W.K., Cooper C.E., Knowles R.G. (2001). Nitric oxide synthases: Structure, function and inhibition. Biochem. J..

[51] Mori M., Gotoh T. (2004). Arginine Metabolic Enzymes, Nitric Oxide and Infection. J. Nutr..

[52] Cusumano Z.T., Watson M.E., Caparon M.G., Camilli A. (2014). *Streptococcus pyogenes* Arginine and Citrulline Catabolism Promotes Infection and Modulates Innate Immunity. Infect. Immun..

[53] Krysenko S., Emani C.S., Bäuerle M., Oswald M., Kulik A., Meyners C., Hillemann D., Merker M., Wohlers I., Hausch F. (2023). GlnA3*_Mt_* is able to glutamylate spermine but it is not essential for the detoxification of spermine in *Mycobacterium tuberculosis*. bioRxiv.

[54] Gamper M., Zimmermann A., Haas D. (1991). Anaerobic regulation of transcription initiation in the *arcDABC* operon of *Pseudomonas aeruginosa*. J. Bacteriol..

[55] Maas W.K. (1994). The arginine repressor of *Escherichia coli*. Microbiol. Rev..

[56] Klingel U., Miller C.M., North A.K., Stockley P.G., Baumberg S. (1995). A binding site for activation by the *Bacillus subtilis* AhrC protein, a repressor/activator of arginine metabolism. Mol. Gen. Genet. MGG.

[57] Rodríguez-García A., Ludovice M., Martín J.F., Liras P. (2003). Arginine boxes and the *argR* gene in *Streptomyces clavuligerus*: Evidence for a clear regulation of the arginine pathway. Mol. Microbiol..

[58] Charlier D., Roovers M., Van Vliet F., Boyen A., Cunin R., Nakamura Y., Glansdorff N., Piérard A. (1992). Arginine regulon of Escherichia coli K-12. J. Mol. Biol..

[59] Tian G., Lim D., Carey J., Maas W.K. (1992). Binding of the arginine repressor of *Escherichia coli* K12 to its operator sites. J. Mol. Biol..

[60] Miller C.M., Baumberg S., Stockley P.G. (2003). Operator interactions by the *Bacillus subtilis* arginine repressor/activator, AhrC: Novel positioning and DNA-mediated assembly of a transcriptional activator at catabolic sites. Mol. Microbiol..

[61] Zeng L., Dong Y., Burne R.A. (2006). Characterization of cis-acting sites controlling arginine deiminase gene expression in *Streptococcus gordonii*. J. Bacteriol..

[62] Marquis R.E., Bender G.R., Murray D.R., Wong A. (1987). Arginine deiminase system and bacterial adaptation to acid environments. Appl. Environ. Microbiol..

[63] Bassoe C.F., Bjerknes R. (1985). Phagocytosis by Human Leukocytes, Phagosomal pH and Degradation of Seven Species of Bacteria Measured by Flow Cytometry. J. Med. Microbiol..

[64] Fulde M., Willenborg J., Huber C., Hitzmann A., Willms D., Seitz M., Eisenreich W., Valentin-Weigand P., Goethe R. (2014). The arginine-ornithine antiporter ArcD contributes to biological fitness of *Streptococcus suis*. Front. Cell. Infect. Microbiol..

[65] Nguyen L.-T., Schmidt H.A., von Haeseler A., Minh B.Q. (2015). IQ-TREE: A Fast and Effective Stochastic Algorithm for Estimating Maximum-Likelihood Phylogenies. Mol. Biol. Evol..

[66] Wu R., Zhao M., Li J., Gao H., Kan B., Liang W. (2015). Direct regulation of the natural competence regulator gene *tfoX* by cyclic AMP (cAMP) and cAMP receptor protein (CRP) in *Vibrios*. Sci. Rep..

[67] Xu Y.W., Xing R.X., Zhang W.H., Li L., Wu Y., Hu J., Wang C., Luo Q.L., Shen J.L., Chen X. (2019). Toxoplasma ROP16I/III ameliorated inflammatory bowel diseases via inducing M2 phenotype of macrophages. World J. Gastroenterol..

[68] Pan J.J., Zhao M., Huang Y.M., Li J., Liu X.S., Ren Z.H., Kan B., Liang W.L. (2018). Integration Host Factor Modulates the Expression and Function of T6SS2 in *Vibrio fluvialis*. Front. Microbiol..

[69] Zianni M., Tessanne K., Merighi M., Laguna R., Tabita F.R. (2006). Identification of the DNA bases of a DNase I footprint by the use of dye primer sequencing on an automated capillary DNA analysis instrument. J. Biomol. Tech..

[70] Monack D.M., Raupach B., Hromockyj A.E., Falkow S. (1996). *Salmonella typhimurium* invasion induces apoptosis in infected macrophages. Proc. Natl. Acad. Sci. USA.

