# Supplementary material for: The ArgR-Regulated ADI Pathway Facilitates the Survival of Vibrio fluvialis under Acidic Conditions
Source: Int J Mol Sci. 2024 May 23;25(11):5679. doi: 10.3390/ijms25115679 (PMC11172107; doi:10.3390/ijms25115679)
Supplement: Supplementary file 1 [file ijms-25-05679-s001.zip › ijms-2960832-Supplementary.pdf]

Table S1 Bacteria strains and plasmids used in this study

Bacterial strains/plasmids	Characteristics	Sources
Escherichia coli		
SM10*λpir*	*thr thi tonA leu supE lacY recA*::RP4-2Tc::Mu （*λpir*R6K）, Km^R	Mekalanos Laboratory (Harvard Medical School)
Top10	F- *mcrA* Δ *(mrr-hsd RMS-mcrBC) φ*80*lacZ*Δ*M*15Δ*lacX*74 *recA*1 *deoRara*Δ139Δ*(ara-leu)*7697 *galU galK rpsL (StrR) endA*1 *nupG*	Laboratory stock
Rosetta (DE3)	F^- *ompT hsdS*$_B$ (r_B^- m_B^-) *gal dcm* (DE3) pRARE2 (Cam^R)	Laboratory stock
Vibrio fluvialis		Laboratory stock
85003	*V. fluvialis*, wild type, Sm^R	Laboratory stock
Δ*arcD*	*arcD* in-frame deletion strain of 85003, Sm^R	This study
Δ*arcDACB*	arc*DACB* in-frame deletion strain of 85003, Sm^R	This study
Δ*argR*	*argR* in-frame deletion strain of 85003, Sm^R	This study
Δ*lacZ*	*lacZ* in-frame deletion strain of 85003, Sm^R	Laboratory stock
Plasmid		
pWM91	suicide vector containing R6 K ori, *sac*B, *lacZ*α; Amp^R	Laboratory stock
pBBR*lux*	promoterless of *lux*CDABE, Cm^R	Laboratory stock
pET30a	expression vector containing f1 ori, *lacI*; Kan^R	Laboratory stock
pWMΔ*arcD*	Δ*arcD* fragment (1423 bp *Xba*I-*Sac*I) of 85003 in pWM91	This study
pWMΔ*arcDACB*	Δ*arcDACB* fragment (5585 bp *Xba*I-*Sac*I) of 85003 in pWM91	This study
pWMΔ*argR*	Δ*argR* fragment (438 bp *Xba*I-*Sac*I) of 85003 in pWM91	This study
p*arcD*-*lux*	promoter region of *arcD* (685 bp) in pBBR*lux*	This study
p*arcACB*-*lux*	promoter region of *arcACB* (852bp) in pBBR*lux*	This study
p*arcD*mu1-*lux*	p*arcD*-*lux* with mutations in the Arg binding site 1	
p*arcD*mu2-*lux*	p*arcD*-*lux* with mutations in the Arg binding site 2	This study
p*arcD*mu3-*lux*	p*arcD*-*lux* with mutations in the Arg binding site 3	This study
p*arcACB*mu1-*lux*	p*arcACB*-*lux* with mutations in the Arg binding site 1	This study
p*arcACB*mu2-*lux*	p*arcACB*-*lux* with mutations in the Arg binding site 2	This study
p*arcACB*mu3-*lux*	p*arcACB*-*lux* with mutations in the Arg binding site 3	This study
p*arcACB*mu4-*lux*	p*arcACB*-*lux* with mutations in the Arg binding site 4	This study
pET*argR*	*argR* ORF (486 bp) of *V. fluvialis* clone in pET30a	This study

Table S2 Primers in this study

Primer name	Sequences(5' ~ 3')	Sources
arc-F1-up-*Xba*I	GCTCTAGATTGAGGTTATCCAGCGTG	Laboratory stock
arc-F1-dn	CTATGACAACGGCGACTAACCCAAAATC	Laboratory stock
arc-F2-up	GTTAGTCGCCGTTGTCATAGCTGTCTCC	Laboratory stock
arc-F2-dn-*Sac*I	CGAGCTCTGTTCGTACTGCCAACCT	Laboratory stock
arcD-F1-up-*Xba*I	GCTCTAGACCTATTTCAGAACCTACG	Laboratory stock
arcD-F1-dn	TATGACAACCGTGCCTTCATGTAAGCAC	Laboratory stock
arcD-F2-up	ATGAAGGCACGGTTGTCATAGCTGTCTC	Laboratory stock
arcD-F2-dn-*Sac*I	GCGAGCTCTTGGCAGCGGTTGAAATG	Laboratory stock
argR-F1-up-XbaI	GCTCTAGAAGGCGGCCTGAAATTGTA	Laboratory stock
argR-F1-dn	CAGTGCCATTCGTCTTGGTGTTGCCTGA	Laboratory stock
argR-F2-up	CACCAAGACGAATGGCACTGTGAGGCAT	Laboratory stock
argR-F2-dn-*Sac*I	CGAGCTCTGGACGAAATCACCACCG	Laboratory stock
arcA-qPCR-up	AAGAATGGCTGCTGAATACG	Laboratory stock
arcA-qPCR-dn	CAGAATAGACGAAGATTGAATGG	Laboratory stock
arcB-qPCR-up	CAACCTGATGCTGCCTTACC	Laboratory stock
arcB-qPCR-dn	GTGTCTTCGCCGTGGAATG	Laboratory stock
arcC-qPCR-up	AACTGGTCGGCGTAGAAG	Laboratory stock
arcC-qPCR-dn	TCATCTGGCGTGGTGTTG	Laboratory stock
arcD-qPCR-up	GCTGCACGCTTTGACCCTG	Laboratory stock
arcD-qPCR-dn	CACAGATGATCCAGCCGACC	Laboratory stock
arc-prom-up-*Sac*I	CGAGCTCAACCTCATCACACCAACG	Laboratory stock
arc-prom-dn-*Bam*HI	CGGGATCCAACGCGACGTAATTGAC	Laboratory stock
arcD-prom-up-*Sac*I	CGAGCTCTTCCTGTTTGGCGGTCTGTT	Laboratory stock
arcD-prom-dn-*Bam*HI	CGGGATCCGGGTTGTCATAGCTGTCTCC	Laboratory stock
argR-up-*Xho*I	CCGCTCGAGGGCAACACCAAGACGCGTTT	Laboratory stock
argR-dn-*Nde*I	CGCCATATGCCTCACAGTGCCATTGCG	Laboratory stock
arcA-race	GCGAGCAAATTAGTGAGTAG	Laboratory stock
arcD-race	ATCACGCCCTGAGGATTACC	Laboratory stock
arcA-shift-up1	GCATACTTCAGTGCTATTGA	Laboratory stock
arcA-shift-up2	CTTCCATAAAATCCGCACAA	Laboratory stock
arcA-shift-dn	AGAGCGATCACAGTTCGATT	Laboratory stock
arcD-shift-up1	GCCACTGAAATAGCAATTGA	Laboratory stock
arcD-shift-up2	CCGCAGGCTCTTTAGAATTT	Laboratory stock
arcD-shift-dn	CCCACACCTCAATTCACTCA	Laboratory stock
p*arcD*-F (FAM)	GTGTCCGATACAGTGACTTG	Laboratory stock
p*arcD*-R	TCAGAGCGGAAACAGAGAAG	Laboratory stock
p*arcACB*-F (FAM)	CATCGGTTTGAGTTTGTCTC	Laboratory stock
p*arcACB*-R	CGTTCTGATATTCATATTACCCC	Laboratory stock
p*lux*-*arcD*-*Sac*I-F	CTCACTATAGGGCGAATTGGAGCTCCTTCCTGTTTGGCG GTCTGTTC	Laboratory stock
arcD-mu1-R	GTGAATAAAAAATAGTGAATTTTGGTTAATTGCTATTTC AG	Laboratory stock

arcD-mu1-F	TGAAATAGCAATTAACCAAAATTCACTATTTTTATTCAC	Laboratory stock
arcD-mu2-R	TGGTGAAAACTTTTAGTGTTGGTTATGGTGATAAAGTG	Laboratory stock
arcD-mu2-F	TCACTTTATCACCATAACCAACACTAAAAGTTTTCACC	Laboratory stock
arcD-mu3-R	TAGAAAACGTTTATTTCAATACTGGTTAATATGGCAACG TATTTC	Laboratory stock
arcD-mu3-F	ATACGTTGCCATATTAACCAGTATTGAAATAAACGTTTT C	Laboratory stock
p*lux*-*arcD*-*Bam*HI-R	CATTTTGCGGCCGCAACTAGAGGATCCGGGTTGTCATA GCTGTCTCC	Laboratory stock
p*lux*-*arcACB*-F-*Sac*I	CTCACTATAGGGCGAATTGGAGCTCTCAACCTCATCAC ACCAACGTC	Laboratory stock
arcACB-mu1-R	CAAATTCATTTTCCGTATTGGTTACTCAATAGCACTGAA G	Laboratory stock
arcACB-mu1-F	TTCAGTGCTATTGAGTAACCAATACGGAAAATGAATTTG	Laboratory stock
arcACB-mu2-R	GAATTGATAGTTGATGAAGTTTGGTTAAATTCATTTTCC	Laboratory stock
arcACB-mu2-F	AAGGAAAATGAATTTAACCAAACTTCATCAACTATCAAT TC	Laboratory stock
arcACB-mu3-R	TGTTTTTTAACAAGGTTTGGTTATCTGGTAGAAGATTAT C	Laboratory stock
arcACB-mu3-F	AATCTTCTACCAGATAACCAAACCTTGTTAAAAAACAC	Laboratory stock
arcACB-mu4-R	GGGATAAATAAAGAATGGTTATGAAATTAGGAGGATG	Laboratory stock
arcACB-mu4-F	ATCCTCCTAATTTCATAACCATTCTTTATTTATCCCG	Laboratory stock
p*lux*-*arcACB*-R-*Bam*HI	CATTTTGCGGCCGCAACTAGAGGATCCAACGCGACGTA ATTGACCTATTTC	Laboratory stock
iNOS-qPCR-up	CACCTTGGAGTTCACCCAGT	[67]
iNOS-qPCR-dn	ACCACTCGTACTTGGGATGC	[67]
ß actin-qPCR-up	TGGAATCCTGTGGCATCCATGAAAC	[67]
ß actin-qPCR-dn	TAAAACGCAGCTCAGTAACAGTCCG	[67]

Table S3 Genetic information of the *arc* gene cluster of 42 *Vibrio* strains

Strain Names	Gene bank Accession	Strain Names	Gene bank Accession
Vibrio mimicus Y4	CP077426.1	*Vibrio tapetis* CECT4600	LT960612.1
V. cholerae N16961	CP028827.1	*Vibrio ishigakensis* JCM 19231	AP024882.1
Vibrio parahaemolyticus RIMD 2210633 substr	NC_004603.1	*Vibrio maritimus* BH16	CP090438.1
Vibrio harveyi ATCC 33843	CP009467.2	*Vibrio mediterranei* 117-T6	CP033578.1
Vibrio alginolyticus ATTCC 17749	CP006718.1	*Vibrio japonicus* JCM 31412	CP102096.1
Vibrio vulnificus FORC_037	CP016321.1	*Vibrio sinaloensis* YA2	CP096199.1
Vibrio aerogenes LMG 19650	AP024861.1	*Vibrio tubiashii* ATCC 19109	NZ_CP009354.1
Vibrio toranzoniae CECT 7225	AP024861.1	*Vibrio europaeus* NPI-1	CP053541.1
Vibrio kanaloae R17	CP065150.1	*Vibrio chagasii* LMG 21353	NZ_AP025465.1
Vibrio qinghaiensis Q67	CP022741.1	*Vibrio artabrorum* CECT 7226	AP025458.1
Vibrio anguillarum PF4-E2-R4	CP031494.1	*Vibrio pomeroyi* LMG 20537	AP025506.1
Vibrio aquimaris THAF100	CP045350.1	*Vibrio gigantis* ACE001	CP092384.1
Vibrio pectenicida LMG 19642	AP024889.1	*Vibrio crassostreae* 9CS106	CP016228.1
Vibrio quintilis CECT 7734	AP024897.1	*Vibrio coralliirubri* DSM 27495	ORXW01000009.1
Vibrio aphrogenes CA-1004 DNA	AP018689.1	*Vibrio celticus* CECT 7224	MVJF01000104.1
Vibrio cortegadensis CECT 7227	AP025472.1	*Vibrio cyclitrophicus* ECSMB14105	CP039700.1
Vibrio metschnikovii 9502-00	CP046793.1	*Vibrio echinoideorum* DSM 107264	AP025483.1
Vibrio gallicus CIP 107863	AP024872.1	*Vibrio splendidus* 2_C04b	CP089203.1
Vibrio furnissii FDAARGOS_777	CP040990.1	*Vibrio lentus* LMG 21034	MCXR02000001.1
Vibrio fluvialis ATCC 33809	CP014035.2	*Vibrio tasmaniensis* LMG 20012	AP025510.1
Vibrio coralliilyticus S2052	CP063051.1	*Vibrio atlanticus* LGP32	FM954972.2

Reference

67. Xu YW, Xing RX, Zhang WH, Li L, Wu Y, Hu J, Wang C, Luo QL, Shen JL, X. C: Toxoplasma ROP16I/III ameliorated inflammatory bowel diseases via inducing M2 phenotype of macrophages. World J Gastroenterol 2019, 25(45):6634-6635.

Figure S1: WT and its mutant strains had no difference in their growth trends under neutral conditions (pH=7)

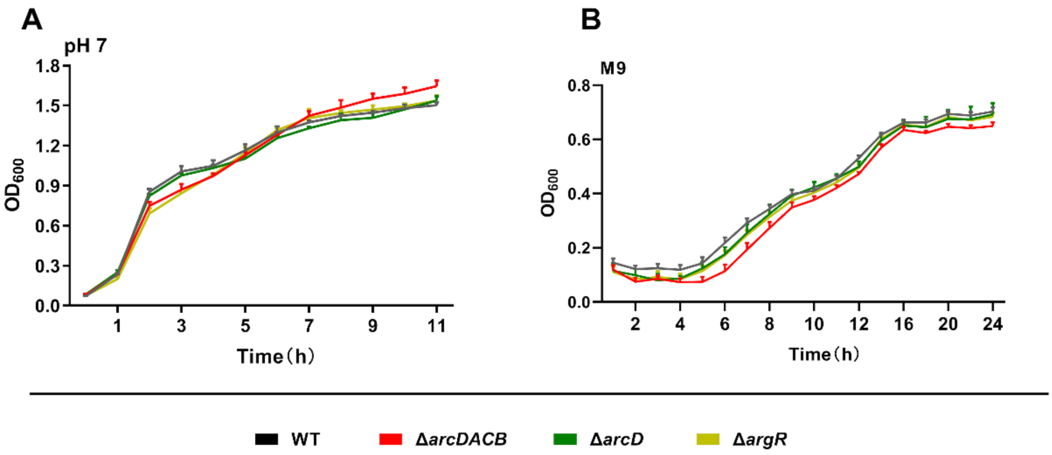

Figure S1: Fresh overnight cultures of the WT and its mutant strains were diluted (1:100) in fresh LB (A) or M9 (B) media at an initial pH of 7 and incubated at 37°C. The OD_{600} was measured at the designated time points.
